# Conditional Knockout of *Pdha1* in Mouse Hippocampus Impairs Cognitive Function: The Possible Involvement of Lactate

**DOI:** 10.3389/fnins.2021.767560

**Published:** 2021-10-14

**Authors:** Wanxin Chen, Xiaoxia Sun, Libin Zhan, Wen Zhou, Tingting Bi

**Affiliations:** ^1^School of Traditional Chinese Medicine & School of Integrated Chinese and Western Medicine, Nanjing University of Chinese Medicine, Nanjing, China; ^2^Centre for Innovative Engineering Technology in Traditional Chinese Medicine, Liaoning University of Traditional Chinese Medicine, Shenyang, China

**Keywords:** PDHA1, knockout mice, hippocampus, cognitive function, lactate

## Abstract

**Background and Purpose:** Neurodegenerative diseases are associated with metabolic disturbances. Pyruvate dehydrogenase E1 component subunit alpha (PDHA1) is an essential component in the process of glucose metabolism, and its deficiency exists in various diseases such as Alzheimer’s disease (AD), epilepsy, Leigh’s syndrome, and diabetes-associated cognitive decline. However, the exact role of PDHA1 deficiency in neurodegenerative diseases remains to be elucidated. In this study, we explored the effect of PDHA1 deficiency on cognitive function and its molecular mechanism.

**Methods:** A hippocampus-specific *Pdha1* knockout (*Pdha1*^–/–^) mouse model was established, and behavioral tests were used to evaluate the cognitive function of mice. Transmission electron microscopy (TEM) was performed to observe the morphological changes of the hippocampus. The lactate level in the hippocampus was measured. Reverse transcription-quantitative polymerase chain reaction (RT-qPCR) and western blotting were used to explore the possible mechanism of the effect of PDHA1 on cognition.

**Results:** *Pdha1* knockout damaged the spatial memory of mice and led to the ultrastructural disorder of hippocampal neurons. Lactate accumulation and abnormal lactate transport occurred in *Pdha1*^–/–^ mice, and the cyclic AMP-protein kinase A-cAMP response element-binding protein (cAMP/PKA/CREB) pathway was inhibited.

**Conclusion:** Lactate accumulation caused by PDHA1 deficiency in the hippocampus may impair cognitive function by inhibiting the cAMP/PKA/CREB pathway.

## Introduction

Metabolic insufficiency occurs in neurodegenerative diseases, including Alzheimer’s disease (AD; Turner, 2021). Pyruvate dehydrogenase complex (PDC) is a crucial enzyme in glucose metabolism, catalyzing the oxidative decarboxylation of pyruvate to acetyl-CoA, which links the cytoplasmic glycolytic pathway with the mitochondrial tricarboxylic acid cycle and oxidative phosphorylation (Patel et al., 2014). Pyruvate dehydrogenase E1 component subunit alpha (PDHA1) is a critical subunit of PDC. The clinical symptoms of PDC deficiency range from fatal lactic acidosis or progressive neuromuscular injury to chronic neurodegeneration (Robinson, 2006; Imbard et al., 2011; Patel et al., 2012; Pavlu-Pereira et al., 2021). These symptoms suggest that the nervous system is susceptible to perturbations in PDC activity due to its dependence on carbohydrate metabolism (Gray et al., 2014). PDHA1 deficiency has been found in a variety of neurodegenerative diseases, such as AD, epilepsy, and Leigh’s syndrome (Gavrilovici and Rho, 2020; Pawlosky et al., 2020; Gong et al., 2021). In addition, our previous study found that the expression of PDHA1 protein is decreased significantly in the hippocampi of rats with diabetes-associated cognitive decline (Shi et al., 2011).

Pyruvate dehydrogenase complex deficiency causes metabolic defects in the brain. On the one hand, the lack of PDC leads to insufficient energy supply to the brain, because the ATP needed by neurons is mainly produced in the mitochondria by oxidative phosphorylation of glucose *via* the tricarboxylic acid cycle (Bordone et al., 2019). On the other hand, PDC deficiency leads to the increase of lactate. In cells lacking PDC, pyruvate produced in the glycolytic pathway cannot enter the tricarboxylic acid cycle, however, it is converted to lactate, resulting in apparent aerobic glycolysis (Biswas et al., 1998). The role of lactate in the brain remains controversial. Lactate, produced from glucose through glycolysis, was initially believed to be a waste product; however, it is now considered an energy substrate and a signaling molecule (Magistretti and Allaman, 2018). Nevertheless, promoting lactate production by genetically manipulating metabolism can lead to oxidative stress and apoptosis in rat cortical neurons (Herrero-Mendez et al., 2009). One study found that lactate levels were elevated with age and correlated with more unsatisfactory memory performance in APP/PS1 mice (Harris et al., 2016). These findings suggest that changes in lactate metabolism may be a factor leading to the decline of cognitive function.

The hippocampus plays a vital role in learning and memory, and its structural and functional changes are associated with the development of neurodegenerative diseases related to cognitive decline (Bettio et al., 2017). Previous studies have not attracted enough attention regarding the effect of PDHA1 deficiency in the hippocampus on cognitive function. In order to solve this problem, we produced a novel hippocampus-specific *Pdha1* knockout (*Pdha1*^–/–^) mouse model. We hypothesized that lactate might be involved in cognitive impairment in *Pdha1*^–/–^ mice through the cyclic AMP-protein kinase A-cAMP response element-binding protein (cAMP/PKA/CREB) signaling pathway.

## Materials and Methods

### Animals

**FIGURE 1:**
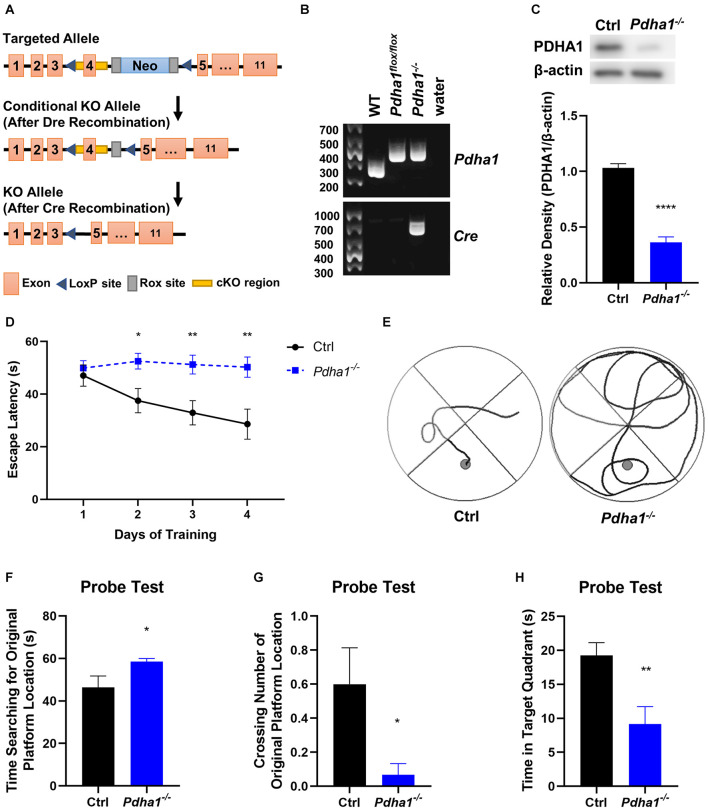
*Pdha1*^–/–^ mice showed spatial memory impairment in the Morris water maze (MWM) test. **(A)** Schematic diagram of *Pdha1* conditional knockout strategy. **(B)** Schematic diagram of PCR genotyping in mouse offspring. The floxed *Pdha1* gene or wild-type gene were amplified as 394 and 281 bp, respectively. Cre was amplified as a 661 bp fragment. **(C)** Representative immunoblots of PDHA1 protein and quantification of PDHA1 protein levels in the hippocampi of *Pdha1*^–/–^ mice and control mice (*n* = 4). **(D)** Escape latencies of the mice in the training trials (*n* = 15). **(E)** Representative swim track of *Pdha1*^–/–^ mice and control mice in the training trials. **(F–H)** The probe test, including time searching for the original platform location **(F)**, number of crossings over the original platform location **(G)**, and time in the target quadrant **(H)** (*n* = 15). All data are expressed as means ± SEM, *p < 0.05, **p < 0.01, ****p < 0.0001.

Seven-month-old, half male and half female mice were used in all experiments. *Pdha1*^–/–^ mice were generated by Cyagen using the strategy outlined in Figure 1A. Eleven exons have been identified in *Pdha1*. Exon 4 was selected as conditional knockout region because deletion of exon 4 should result in the loss of function of the *Pdha1* gene. In the targeting vector, the Neo cassette was flanked by Rox sites, and cKO region was flanked by LoxP sites. The conditional KO allele was obtained after Dre-mediated recombination. The KO allele was obtained after Cre-mediated recombination. Figure 1B illustrates the genotyping strategy. For more information on genotyping, please see the Supplementary Material. The mice carrying KO allele were identified by genotyping, which were named *Pdha1*^–/–^ mice and used in all experiments. Control wild-type mice were purchased from Vital River Laboratories (China). All mice were on a C57BL/6N background. Mice were raised in the temperature-controlled (24 ± 2°C) specific pathogen-free animal experiment center of Nanjing University of Chinese Medicine, accepted a 12 h light/dark cycle with free access to food and water. All animal experiments were approved by the Animal Ethics Committee of Nanjing University of Chinese Medicine (Approval No. 201812A028).

### Behavioral Tests

We used a series of behavioral tests, including the open field test (OFT), the novel object recognition (NOR) test, and the Morris water maze (MWM) test. The tests were performed on the following schedule: day 1, the OFT; day 2, the NOR test; and days 3–7, the MWM test.

#### Open Field Test

The OFT assessed spontaneous locomotor in mice. The test was performed in a 25 cm × 25 cm × 35 cm box. Mice were placed in the center of the box and allowed to explore freely for 5 min. Behavioral patterns measured included total distance, velocity, time in the center (5 cm × 5 cm), and entries in the center. The locomotion was recorded by video camera and registered on the computer. The square arena was cleaned with 75% alcohol after each trial.

#### Novel Object Recognition Test

The NOR test is used to evaluate short-term memory in mice (Pałucha-Poniewiera et al., 2021). First, a mouse was placed in a 25 cm × 25 cm × 35 cm box to adapt to 5 min. Next, the mice were presented with two objects, A and B, for 5 min. One hour later, we replaced object B with a novel object C and repeated the trial. The square arena was cleaned with 75% alcohol after each trial. The interaction time with the novel object (T_C_) and familial object (T_A_) was recorded. The discrimination index is defined as T_C_/(T_A_ + T_C_), which indicated new object recognition ability.

#### Morris Water Maze Test

The MWM test evaluated the spatial learning and memory performance of mice. The water maze consisted of a pool (120 cm in diameter and filled with water maintained at 24 ± 1°C) and a platform (9 cm in diameter) submerged 1 cm under the water. On the first day, mice were put into the pool to swim freely for 60 s to adapt to the new condition without the platform before trials. During the 4-day training trial, mice were allowed to swim for 60 s to locate the hidden platform and rest for 60 s upon finding the platform. If the mouse failed to reach the platform within 60 s, it was gently guided to the platform and allowed to stay for 60 s. Mice were trained with four trials per day. The escape latency taken to reach the platform was measured automatically. Finally, the hidden platform was removed for the probe trial test. The time searching for the original platform, the number of platform crossings, and time spending in the target quadrant (the quadrant where the platform was located) were recorded. All data were measured by a camera and automated analysis system.

### Transmission Electron Microscopy

Hippocampus samples were fixed with 2.5% glutaraldehyde. After rinsing with 0.1 M phosphoric acid rinse solution, samples were placed in 1% osmium acid and fixed at 4°C for 2 h. Samples were dehydrated in a graded series of ethanol, embedded, cured, and sliced into thin sections (70 nm) with an ultramicrotome (EM UC6, Leica, Germany). After staining with 3% uranyl acetate and lead citrate, the samples were observed with the transmission electron microscope (JEM1230, JEOL, Japan).

### Lactate Measurement

The lactate concentration of hippocampus samples was measured with Amplite^TM^ Colorimetric L-Lactate Assay Kit (13815, AAT Bioquest, United States). The reaction was incubated at room temperature for 2 h, protected from light, and the increase in absorbance ratio was detected at A575 nm/A605 nm. The data were analyzed according to the manufacturer’s instructions.

### Western Blotting

Hippocampus tissues were homogenized in RIPA buffer (P0012B, Beyotime, China) with a protease inhibitor cocktail (5871S, CST, United States) and phosphatase inhibitor cocktail (5870S, CST, United States). Extracted proteins were separated by 10% Sodium Dodecyl Sulfate-Polyacrylamide Gel Electrophoresis (SDS-PAGE) and transferred to PVDF membranes, and immunoblotted with the following antibodies: Pyruvate dehydrogenase E1 component subunit alpha (PDHA1) (sc-377092, Santa Cruz, United States, 1:1000), Monocarboxylate transporter 1 (MCT1) (20139-1-AP, Proteintech, China 1:10000), Monocarboxylate transporter 2 (MCT2) (sc-166925, Santa Cruz, United States,1:1000), Monocarboxylate transporter 4 (MCT4) (sc-376140, Santa Cruz, United States, 1:1000), phospho-Protein kinase A (PKA) (T197) (ab75991, Abcam, United Kingdom, 1:5000), PKA (sc-390548, Santa Cruz, United States, 1:1000), phospho-Cyclic AMP-responsive element-binding protein (CREB) (Ser133) (sc-81486, Santa Cruz, United States, 1:1000), CREB (sc-240, Santa Cruz, United States, 1:1000), HCAR1 (DF2766, Affinity, United States, 1:1000), and β-actin (3700S, CST, United States, 1:1000). The membranes were then treated with HRP-IgG antibody (BA-1054/BA1050, Boster, China, 1:2000). Antibody-bound protein bands were treated with a chemiluminescence solution (ECL, Tanon, China), and proteins were detected using an Amersham Imager 600 (General Electric Company, United States). Band intensities were analyzed with ImageQuant TL 1D software (GE Healthcare, United States).

### Reverse Transcription-Quantitative Polymerase Chain Reaction

Total RNA of hippocampus samples was extracted using RNApure Tissue&Cell Kit (CW0584S, Cwbio, China). The total RNA was converted to cDNA using PrimeScript RT Master Mix (Perfect Real Time) (RR036A, Takara, Japan) and the following reaction conditions: 37°C for 15 min, 85°C for 15 s, and 4°C for 10 min. qPCR was conducted using SYBR Green Master Mix (Q141-02, Vazyme, China), and the reaction program used as follows: 95°C for 5 min, 95°C for 10 s, and 60°C for 30 s, and 95°C for 15 s, 60°C for 60 s, and 95°C for 15 s. Data were analyzed by the ΔΔCt method using β-actin as the reference gene. The primers were designed as follows: PKA, forward 5′-AGATCGTCCTGACCTTTGAGT-3′ and reverse 5′-GGCAAAACCGAAGTCTGTCAC-3′; CREB, forward 5′-AGCAGCTCATGCAACATCATC-3′ and reverse 5′-AGTCCTTACAGGAAGACTGAACT-3′; β-actin, forward 5′-GGCTGTATTCCCCTCCATCG-3′ and reverse 5′-CCAGTTGGTAACAATGCCATGT-3′.

### Statistical Analysis

GraphPad Prism 8.3 statistical software (GraphPad Software, United States) was used for statistical analysis. All data are expressed as mean ± standard error of mean (SEM). Statistical analyses were performed using Student’s *t*-tests for comparing two groups. Training curves were analyzed by two-way repeated-measure ANOVAs and Sidak’s multiple comparison tests. *p* < 0.05 was considered statistically significant.

## Results

### *Pdha1*^–/–^ Mice Show Spatial Memory Impairment

To explore the effects of PDHA1 on cognitive function, we used the strategy shown in Figure 1A to generate *Pdha1*^–/–^ mice. DNA prepared from tail snips of the knockout mice was analyzed by PCR, and the offspring of the dominant *Pdha1* gene (394 bp) and *Cre* gene (661 bp) were *Pdha1*^–/–^ mice (Figure 1B). The hippocampi of mice were extracted to evaluate the expression of PDHA1. As expected, we observed that the expression of PDHA1 in the hippocampal protein extracts from *Pdha1*^–/–^ mice decreased by ∼65% (Figure 1C). This result demonstrates that we successfully established a mouse model where the expression of PDHA1 is significantly reduced in the hippocampus.

Spatial memory was evaluated with the MWM test. In the training test, *Pdha1*^–/–^ mice typically took longer to reach the platform during the 4-day training period than the control mice (Figure 1D). At the same time, the trajectory map of *Pdha1*^–/–^ mice was disorganized and purposeless (Figure 1E). In the space probe test, we found that *Pdha1*^–/–^ mice spent more time searching for the original platform location (Figure 1F). Compared with the control group, the crossing number of the original platform and the time spent in the target quadrant were significantly reduced in *Pdha1*^–/–^ mice (Figures 1G,H). Overall, *Pdha1*^–/–^ mice showed spatial learning and memory impairment.

### *Pdha1*^–/–^ Mice Do Not Show Obvious Spontaneous Locomotor Activity Changes or Novel Object Recognition Impairments

**FIGURE 2:**
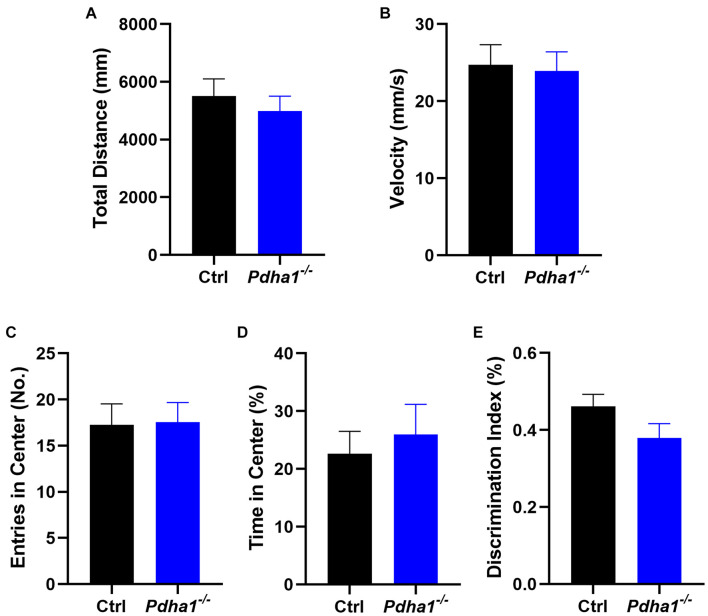
*Pdha1*^–/–^ mice showed normal spontaneous locomotor activity and novel object recognition (NOR) memory. **(A–D)** Open field test (OFT), including total distance **(A)**, velocity **(B)**, entries in the center **(C)**, and time in the center **(D)** (*n* = 15). **(E)** Discrimination index in the novel object recognition (NOR) test (*n* = 15). All data are expressed as means ± SEM.

To assess whether the behavioral changes of *Pdha1*^–/–^ mice were associated with spontaneous locomotor impairments, the OFT was carried out (Figures 2A–D). Behavior parameters, including total distance (Figure 2A), velocity (Figure 2B), entries in the center (Figure 2C), and time in the center (%) (Figure 2D), were not significantly different between *Pdha1*^–/–^ mice and control mice, indicating that the impaired behavioral performance in *Pdha1*^–/–^ mice was not caused by reduced locomotor ability. In the NOR test, the recognition index of *Pdha1*^–/–^ mice was slightly lower than that of control mice, but the difference was not significant (Figure 2E). Together, these results indicated normal spontaneous locomotor activity and novel object identification memory in *Pdha1*^–/–^ mice.

### *Pdha1*^–/–^ Mice Display Abnormal Ultrastructure Morphology in Hippocampal Neurons

**FIGURE 3:**
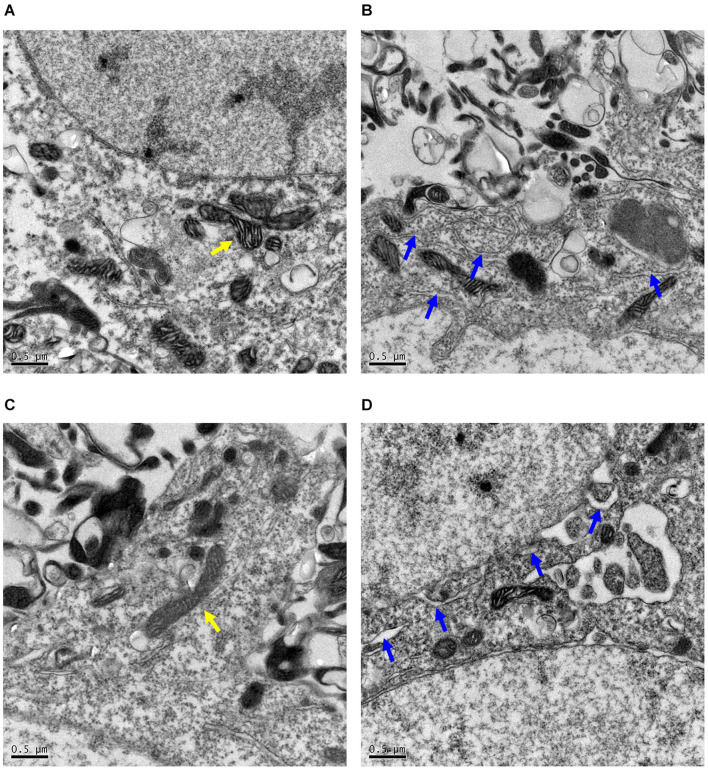
Representative images of hippocampal neurons morphological changes evaluated by transmission electron microscopy (TEM). **(A,B)** The mitochondria and endoplasmic reticulum are structurally intact in control mice (*n* = 2). **(C,D)** By contrast, endoplasmic reticulum was dilatated and vesiculated, and mitochondria cristae appear vague in *Pdha1*^–/–^ mice (*n* = 2). Yellow arrows indicate mitochondria, and blue arrows indicate endoplasmic reticulum. Scale bars = 0.5 μm.

We analyzed the organelle structure of the hippocampal neurons in mice by transmission electron microscopy (TEM). Healthy mitochondria and endoplasmic reticulum ultrastructure were observed in control mice (Figures 3A,B). However, in *Pdha1*^–/–^ mice, the structure of the mitochondrial crest was disordered (Figure 3C), which is a typical feature of dysfunctional mitochondria. Moreover, the rough endoplasmic reticulum was dilated, vesiculated, and partially degranulated in hippocampal neurons of *Pdha1*^–/–^ mice (Figure 3D). The results showed that the ultrastructure of hippocampal neurons in *Pdha1*^–/–^ mice was damaged.

### *Pdha1*^–/–^ Mice Show Increased Lactate Levels and Abnormal Lactate Transport in the Hippocampus

**FIGURE 4:**
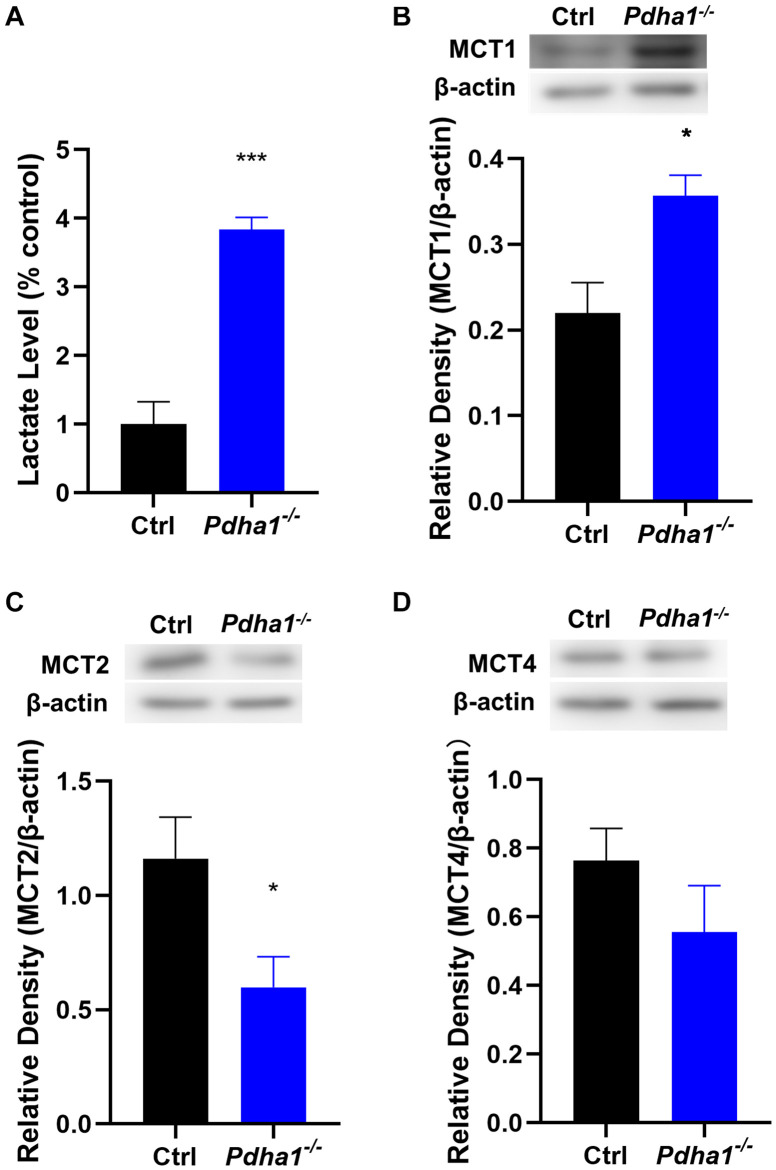
*Pdha1*^–/–^ mice showed increased lactate levels and MCT expressions were changed in the hippocampus. **(A)** Quantification of lactate levels. **(B)** Representative immunoblots of MCT1 protein and quantification of MCT1 protein levels in the hippocampi of *Pdha1*^–/–^ mice and control mice (*n* = 4). **(C)** Representative immunoblots of MCT2 protein and quantification of MCT2 protein levels in the hippocampi of *Pdha1*^–/–^ mice and control mice (*n* = 4). **(D)** Representative immunoblots of MCT4 protein and quantification of MCT4 protein levels in the hippocampi of *Pdha1*^–/–^ mice and control mice (*n* = 4). All data are expressed as means ± SEM, *p < 0.05, ***p < 0.001.

Lactic acidosis following glycolysis caused by decreased PDC activity is a key step related to metabolic reprogramming in the development of neurological disorders (Park et al., 2018). So, we first measured lactate levels in the hippocampus and found a significant increase in *Pdha1*^–/–^ mice (Figure 4A). The transmembrane movement of lactate to or from cells is mainly mediated by monocarboxylate transporters (MCTs; Jha et al., 2016). In the brain, MCT1 is mainly expressed in endothelial cells and astrocytes. MCT2 mediates lactate uptake and is mainly expressed in neurons. MCT4 is expressed in astrocytes and plays a role in lactate output (Deitmer et al., 2019). Thus, we further examined the protein expression of MCTs. The results showed that, compared with the control group, the levels of MCT1 in *Pdha1*^–/–^ mice increased significantly (Figure 4B). Conversely, we observed a significant decrease in MCT2 levels (Figure 4C). However, there were no significant differences in MCT4 levels between *Pdha1*^–/–^ mice and control mice (Figure 4D). These results suggested that the decline of cognitive function in *Pdha1*^–/–^ mice may be related to abnormal lactate metabolism.

### *PDHA1* Deficiency Inhibits the PKA/CREB Signaling Pathway

**FIGURE 5:**
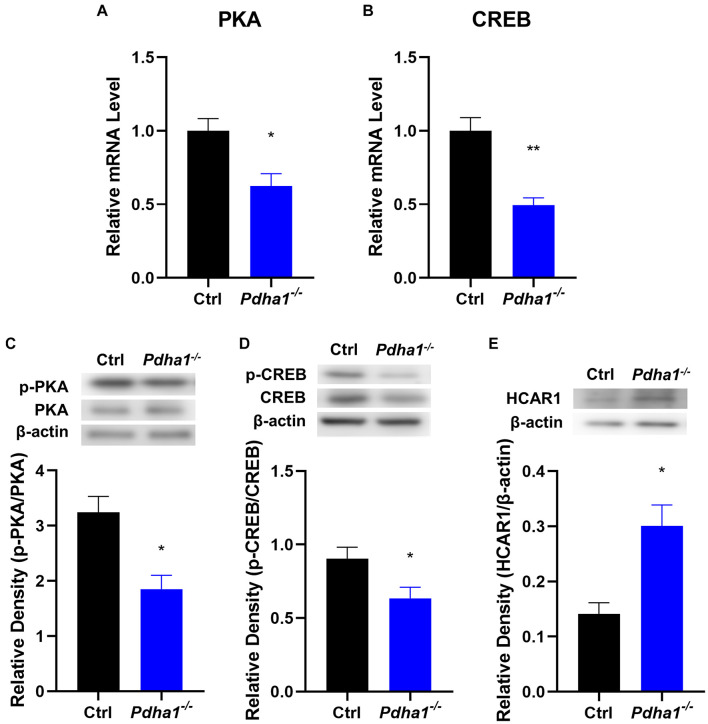
*Pdha1*^–/–^ mice showed an inhibited PKA/CREB signaling pathway. **(A,B)** RT-qPCR determination of mRNA levels of PKA and CREB in hippocampi from *Pdha1*^–/–^ mice and control mice (*n* = 4). **(C)** Representative immunoblots of p-PKA and PKA protein and quantification of p-PKA/PKA protein levels in the hippocampi of *Pdha1*^–/–^ mice and control mice (*n* = 4). **(D)** Representative immunoblots of p-CREB and CREB protein, and quantification of p-CREB/CREB protein levels in the hippocampi of *Pdha1*^–/–^ mice and control mice (*n* = 4). **(E)** Representative immunoblots of HCAR1 protein and quantification of HCAR1 protein levels in the hippocampi of *Pdha1*^–/–^ mice and control mice (*n* = 4). Data are expressed as means ± SEM, *p < 0.05, **p < 0.01.

The hippocampus is a critical tissue that regulates cognitive function, and the cAMP/PKA/CREB pathway plays an important role in brain function, so we analyzed the expression of PKA and CREB in the hippocampi of *Pdha1*^–/–^ mice. The results showed significantly decreased PKA and CREB mRNA levels in *Pdha1*^–/–^ mice (Figures 5A,B). Compared with the age-matched control group, the phosphorylation activities of PKA and CREB in *Pdha1*^–/–^ mice were significantly inhibited (Figures 5C,D), indicating that PKA/CREB transduction was inhibited under the condition of PDHA1 deficiency. We speculated that the lactate accumulation could downregulate the cAMP/PKA/CREB signaling pathway because the lactate activates HCAR1, so we detected the protein expression of HCAR1 in the hippocampi of *Pdha1*^–/–^ mice. As we expected, the expression of HCAR1 in *Pdha1*^–/–^ mice increased significantly (Figure 5E).

## Discussion

**FIGURE 6:**
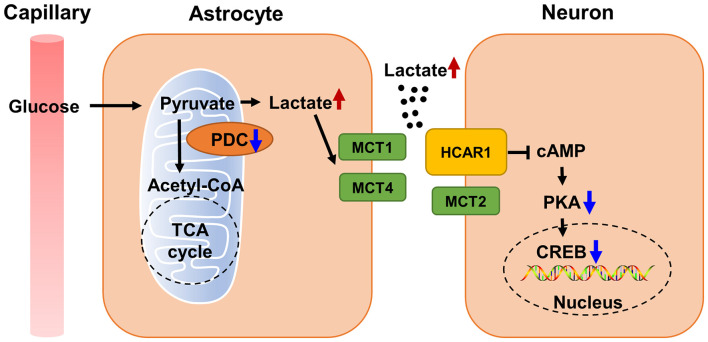
Schematic diagram of cognitive function impaired by abnormal lactate metabolism in *Pdha1*^–/–^ mice. Due to decreased PDC activity, lactate abnormally increases, and excessive lactate transport through MCTs leads to tissue microenvironment acidification. Increased lactate also inhibits the cAMP/PKA/CREB signaling pathway by activating HCAR1, resulting in the decline of cognitive function in mice.

Although there is substantial evidence that PDHA1 deficiency is closely related to neurodegenerative diseases and cognitive impairment, it is unclear whether the loss of PDHA1 in the hippocampus will harm cognitive function. We used *Pdha1*^–/–^ mice to resolve this issue. Our current studies confirmed that PDHA1 deficiency in the hippocampus can lead to spatial memory impairment and ultrastructural damage to hippocampal neurons. In this case, the aerobic oxidation of glucose downstream of PDHA1 was blocked, which leads to the abnormal increase of the metabolite lactate. In addition, lactate transport was changed, which altered lactate homeostasis in the hippocampus. Elevated lactate may not only lead to tissue microenvironment acidification, but also act as a signaling molecule to inhibit the cAMP/PKA/CREB signaling pathway, thus hindering the formation of long-term memory (Figure 6). In conclusion, our study illustrates the important role of PDHA1 in learning and memory, and it may be a potential target in the treatment of cognitive decline.

In order to study whether hippocampal PDHA1 deficiency affects learning and memory, we conducted a series of behavioral tests on animals. The MWM test was used to evaluate the spatial learning and memory ability of mice. When animals cannot find the platform in the water maze, they cannot remember the spatial information they should have obtained during the training days, reflecting impairment of hippocampal-dependent spatial cognition (Kim et al., 2016). Our data showed impaired spatial learning and memory abilities in *Pdha1*^–/–^ mice, suggesting that hippocampal PDHA1 deficiency can lead to hippocampal dysfunction. It is well known that the hippocampus plays an essential role in storing and processing spatial information and transforming short-term memory into long-term memory. Interestingly, the short-term memory of the tested mice was not disturbed in the NOR test, indicating that the loss of PDHA1 in the hippocampus does not affect short-term memory. Furthermore, metabolic dysfunction caused by PDC deficiency can result in defective mitochondrial morphology (Perez-Siles et al., 2016; Park et al., 2018). We found that the deletion of *Pdha1* led to the destruction of the ultrastructure of neurons in the hippocampus. To sum up, we concluded that the deletion of *Pdha1* affected spatial memory and the ultrastructure of neurons.

In order to further explain the possible mechanism of cognitive impairment caused by the loss of PDHA1, we measured the lactate levels in the hippocampi of mice. The results showed increased lactate levels in *Pdha1*^–/–^ mice. Similar to our results, *Pdha1* knockout tumor cells show an increase in extracellular acidification rate, an increase in extracellular lactate levels, and a decrease in ATP production, indicating that *Pdha1*-deleted cells cannot perform normal mitochondrial oxidative phosphorylation but are forced to undergo glycolysis (Zhong et al., 2017; Liu et al., 2019). The uptake of lactate by neurons is vital for establishing long-term memories (Camandola and Mattson, 2017). However, some researchers believe that excessive lactate impairs the function of neurons (Kweon and Suh, 2013; Scandella and Knobloch, 2019). The increase of lactate concentrations in the brain is usually related to brain diseases with cognitive impairments, such as leukoencephalopathy and AD (Scheper et al., 2007; Liguori et al., 2015; Cunnane et al., 2020). One study has shown that continuous lactate accumulation contributes to impaired adult neurogenesis, indicating that extracellular lactate levels should be strictly regulated (Wang et al., 2019). In summary, lactate may benefit the nervous system under physiological conditions, but the increase of pathological lactate may damage cognitive function.

There is evidence that the decrease of extracellular pH is accompanied by increased tissue lactate (Schneider et al., 1993). When lactate is produced, an essential way to acidify the extracellular environment is through MCTs (Spencer and Stanton, 2019). MCT1–MCT4 catalyze proton-coupled transport of lactate (Halestrap and Price, 1999). Brain cells rely on MCTs to maintain pH homeostasis, and the normal function of MCTs can prevent intracellular lactate accumulation and acidosis (Zhang et al., 2020). Our study found that the expression of MCT1 increased and MCT2 decreased in the hippocampi of *Pdha1*^–/–^ mice, which may be an adaptation to the higher concentrations of lactate produced by glycolysis. The excess lactate produced in hippocampal cells of *Pdha1*^–/–^ mice needs to be excreted by MCT1, which may lead to the compensatory increase of MCT1. MCT-mediated H^+^ efflux exacerbates extracellular acidification (Ames et al., 2018). Normally, neurons absorb lactate through MCT2 as a source of energy (Kennedy and Dewhirst, 2010), whereas lactate utilization still requires the normal expression of PDHA1. The expression of MCT2 was downregulated in *Pdha1*^–/–^ mice. One possible explanation is that neurons cannot utilize lactate as an energy substrate due to the loss of PDHA1. In summary, our results confirmed that lactate homeostasis in the hippocampi of *Pdha1*^–/–^ mice was disrupted.

Lactate can be used as a direct agonist of hydroxycarboxylic acid receptor 1 (HCAR1, also known as GPR81) to participate in intercellular signal transduction (Lauritzen et al., 2014; Morland et al., 2017). The cAMP/PKA/CREB pathway is closely related to learning and memory. HCAR1 activation downregulates cAMP and weakens the signal transduction mediated by PKA (Langin, 2010). Activated PKA leads to phosphorylation of CREB, which initiates the transcription and translation of CREB target genes (Lonze and Ginty, 2002). CREB is a transcription factor that regulates neuronal growth, neuronal differentiation, neurogenesis, maturation of neurons, synaptic plasticity, spatial memory, and long-term memory (Sharma and Singh, 2020). Dysfunction of CREB is strongly correlated with several neurodegenerative diseases, including AD, Parkinsonism, Huntington’s disease, and ischemia (De Felice et al., 2007). Our study found that *Pdha1* knockout inhibited the cAMP/PKA/CREB signaling pathway, which may be mediated by lactate. Similar to our results, some studies have shown that lactate inhibits the HCAR1/PKA/CREB pathway in the brain of diabetes-associated cognitive decline rats (Dong et al., 2018; Zhao et al., 2018).

In conclusion, our results suggest that *Pdha1* knockout in the hippocampus leads to the accumulation of lactate and impairs gene expression behind learning and memory, resulting in memory and cognitive impairment. These results may help us understand the pathologies of memory or cognitive impairment, including neurodegenerative conditions such as AD, diabetes-associated cognitive decline, and dementia.

This study has several limitations. First, we only studied the effect of PDHA1 deficiency on lactate, the final product of glycolysis, but the conversation of pyruvate to acetyl-CoA, glycolysis and TCA cycle remains to be further clarified. Second, we did not compare the degree of decline in expression of PDHA1 in different cell types in this mouse model. We hope to knock out *Pdha1* in different cell types in the future to clarify the more detailed functions and mechanisms of PDHA1. Third, we only used 7-month-old mice for the study. Of note, neurodegenerative diseases are common chronic diseases that are associated with age (Hou et al., 2019). In the future, we will include mice of different months in the study to explore the relationship between cognitive impairment and age in *Pdha1*^–/–^ mice.

## Data Availability Statement

The original contributions presented in the study are included in the article/Supplementary Material, further inquiries can be directed to the corresponding author/s.

## Ethics Statement

The animal study was reviewed and approved by the Animal Ethics Committee of Nanjing University of Chinese Medicine.

## Author Contributions

LZ conceived the idea, directed the project, designed the experiments, and involved in modifying the manuscript. WC and XS performed the experiments and analyzed the data. WC wrote the manuscript. LZ, XS, WZ, and TB edited the manuscript. All authors contributed to manuscript revision, read, and approved the submitted version.

## Conflict of Interest

The authors declare that the research was conducted in the absence of any commercial or financial relationships that could be construed as a potential conflict of interest.

## Publisher’s Note

All claims expressed in this article are solely those of the authors and do not necessarily represent those of their affiliated organizations, or those of the publisher, the editors and the reviewers. Any product that may be evaluated in this article, or claim that may be made by its manufacturer, is not guaranteed or endorsed by the publisher.
