# Supplementary material for: Conditional Knockout of Pdha1 in Mouse Hippocampus Impairs Cognitive Function: The Possible Involvement of Lactate
Source: Front Neurosci. 2021 Oct 14;15:767560. doi: 10.3389/fnins.2021.767560 (PMC8552971; doi:10.3389/fnins.2021.767560)
Supplement: Supplementary file 1 [file Image_1.pdf]

Supplementary Material

1 Supplementary Figures

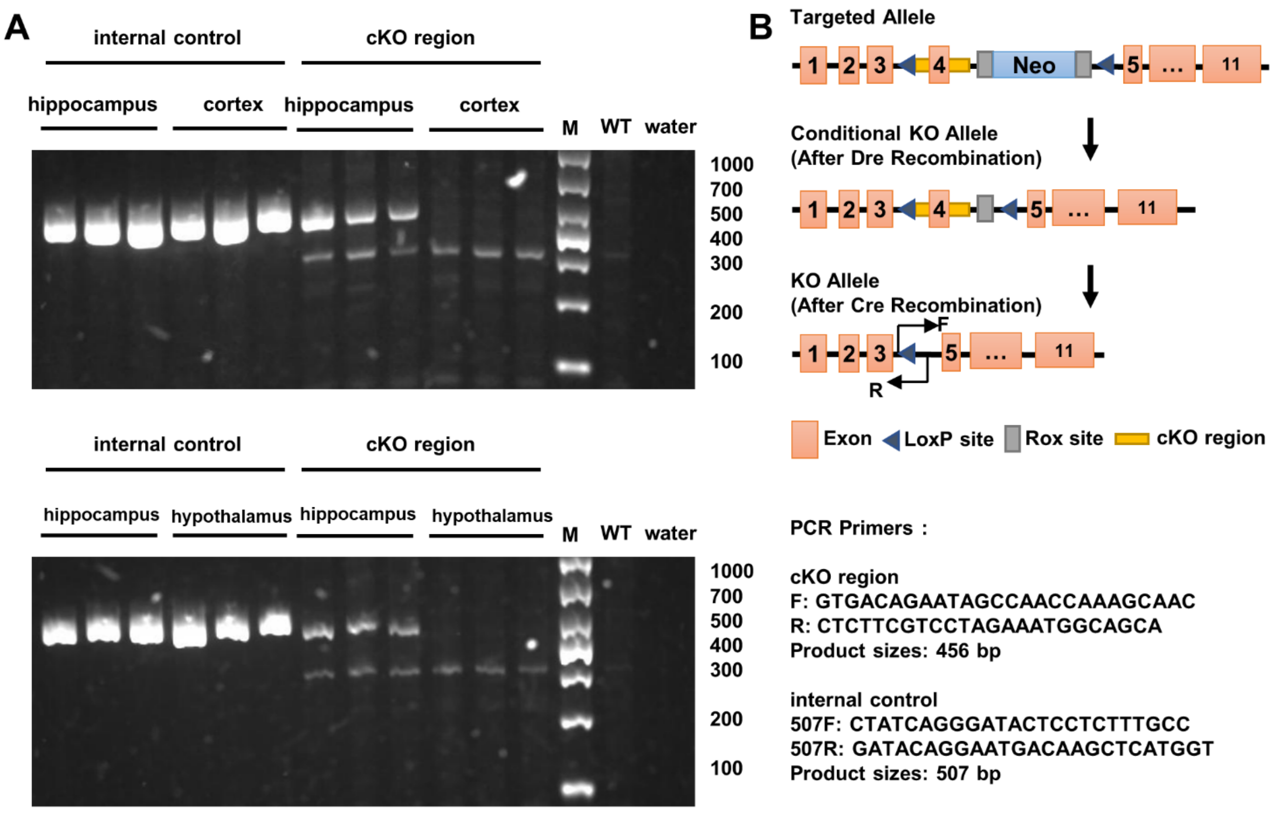

Supplementary Figure 1. *Pdha1* knockout occured in the hippocampus rather than in the cortex and hypothalamus. (A) Genotyping of *Pdha1*$^{-/-}$ mice by PCR analysis. The target bands appeared at 456 bp only in the hippocampus ($n = 3$). **(B)** The information of PCR primers.

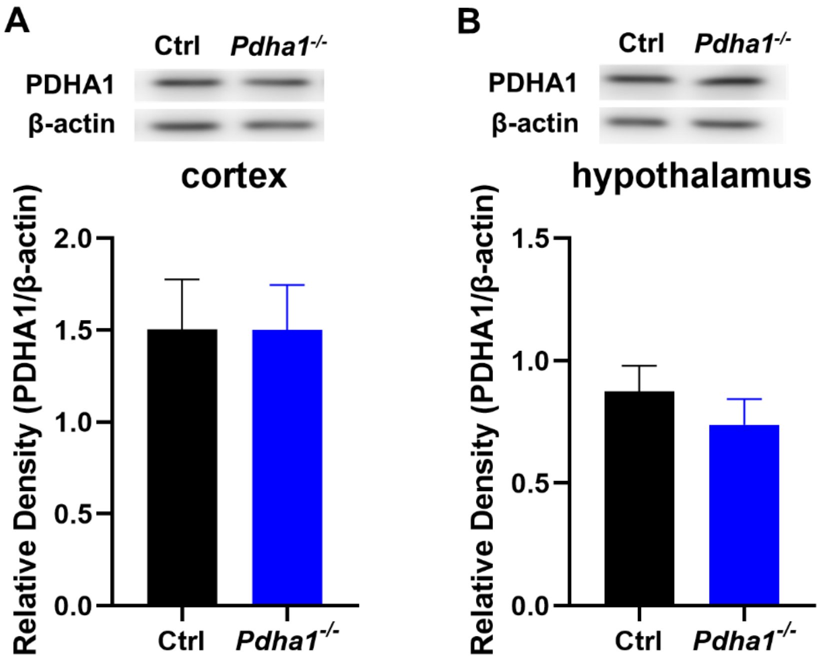

Supplementary Figure 2. There was no change in the expression of PDHA1 protein in cortex and hypothalamus of *Pdha1*$^{-/-}$ mice. (A) Representative immunoblots of PDHA1 protein and quantification of PDHA1 protein levels in the cortex of *Pdha1*$^{-/-}$ mice and control mice (n = 4). **(B)** Representative immunoblots of PDHA1 protein and quantification of PDHA1 protein levels in the hypothalamus of *Pdha1*$^{-/-}$ mice and control mice (n = 4). All data are expressed as means ± SEM.

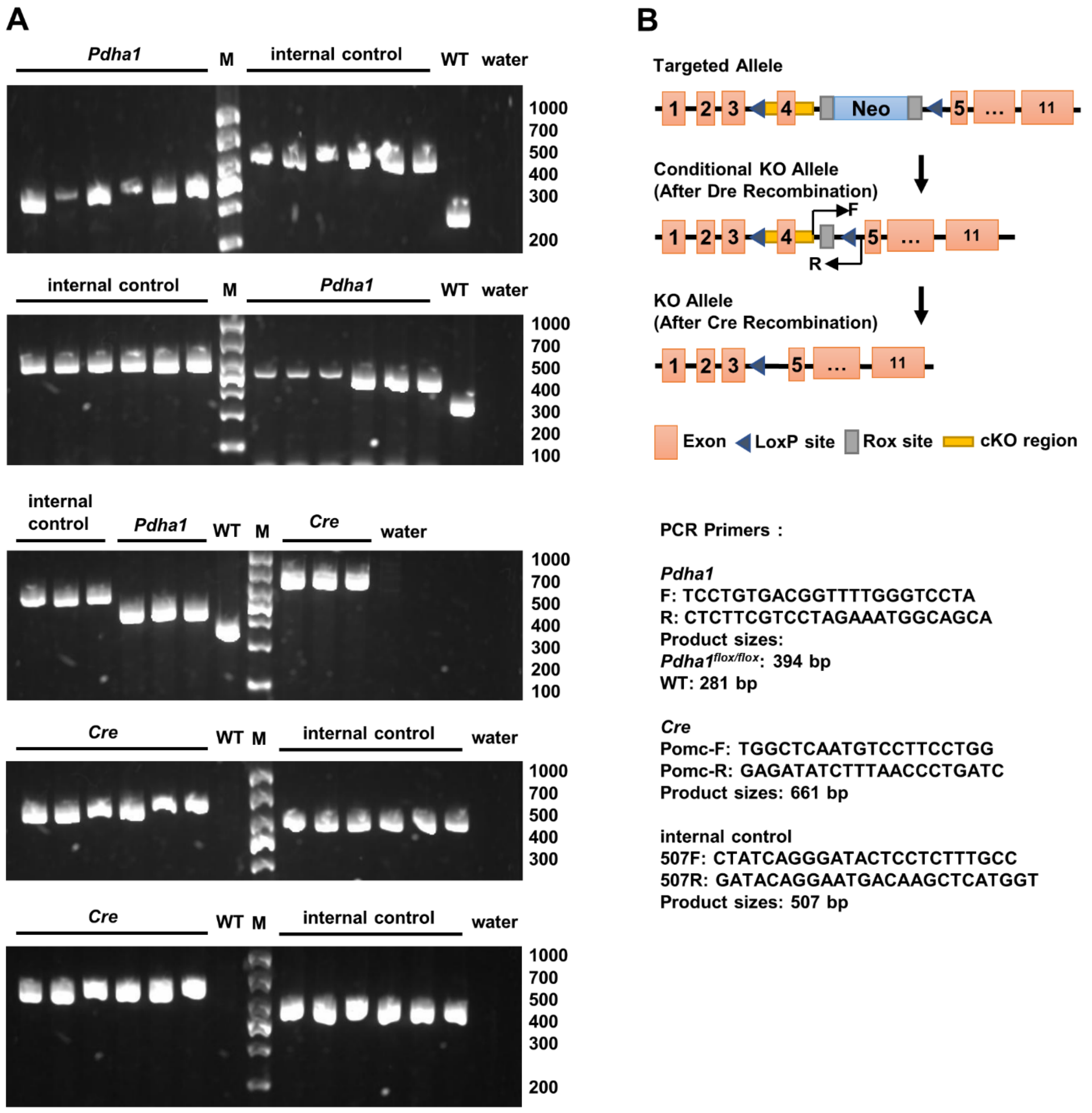

Supplementary Figure 3. The genotyping results of all *Pdha1*$^{-/-}$ mice. (A) Genotyping of *Pdha1*$^{-/-}$ mice by PCR analysis (*n* = 15). **(B)** The information of PCR primers.
